# A Modified Transverse Process-Pedicle Approach Applied to Unilateral Extrapedicular Percutaneous Vertebroplasty

**DOI:** 10.1155/2021/6493712

**Published:** 2021-10-22

**Authors:** Yunyun Zhuo, Liehua Liu, Haoming Wang, Pei Li, Qiang Zhou, Yugang Liu

**Affiliations:** ^1^Department of Orthopedics, The Third Affiliated Hospital of Chongqing Medical University, Chongqing 400010, China; ^2^Department of Orthopedics, Three Gorges Central Hospital, Chongqing 404000, China; ^3^Department of Spine Surgery, Ninth People's Hospital of Chongqing, Chongqing 400700, China

## Abstract

**Objective:**

To introduce a modified transverse process-pedicle puncture technique applied to unilateral extrapedicular percutaneous vertebroplasty (PVP) for the treatment of osteoporotic lumbar vertebral compression fractures.

**Methods:**

A retrospective study was performed on 91 patients with osteoporotic vertebral compression fractures (OVCFs) who underwent unilateral extrapedicular PVP from June 2016 to September 2018. Lumbar and back pain was assessed through the visual analogue scale (VAS). Function recovery was assessed through the Oswestry disability index (ODI). Radiologic outcomes were assessed mainly on the basis of bone cement distribution and anterior vertebral height.

**Results:**

A total of 101 fractured vertebrae were successfully treated using the extrapedicular technique without any recognized clinical complications. The postoperative VAS and ODI values were significantly lower than the corresponding preoperative values (*P* < 0.01). Radiologic outcomes in all fractured vertebrae showed that the diffusion of bone cement could exceed the midline of the vertebral body. There was no significant difference between preoperative and postoperative anterior vertebral heights (*P* < 0.05).

**Conclusion:**

The modified transverse process-pedicle approach applied to unilateral extrapedicular percutaneous vertebroplasty is a simple, safe, and effective surgical method.

## 1. Introduction

Osteoporotic vertebral compression fractures (OVCFs) are one of the most common complications of osteoporosis in the elderly and often lead to severe back pain, kyphosis, impaired mobility, and reduced quality of life [1–3]. Currently, percutaneous vertebroplasty (PVP) is a widely used procedure for the clinical treatment of OVCFs and can obviously relieve pain, reduce bed rest time, and prevent deformity due to collapse of the vertebral body [4–6]. It is generally known that PVP through a bilateral transpedicular approach is the classic procedure performed to treat OVCFs [7, 8]. In recent years, unipedicular PVP has been advocated, reducing the operation and radiation exposure time periods and lowering the risk of cement leakage and complications caused by vertebral pedicle puncture [2, 9, 10]. However, due to the large sagittal diameter of the spinal canal, the long pedicle length, and the small angle of the pedicle in the coronal position, it is difficult to achieve proper bilateral diffusion through unilateral pedicle puncture in the treatment of lumbar vertebral compression fractures. In addition, it is difficult to detect cement diffusion in bilateral puncture, which increases the risk of this surgery. In this article, we will demonstrate a simple and easy unilateral puncture method for extrapedicular PVP and show that it has the advantages of safety, efficiency, and less pain.

## 2. Materials and Methods

Before surgery, informed consent was obtained from all patients after fully explaining the treatment process. This study was approved by the medical ethics committee of the Third Affiliated Hospital of Chongqing Medical University.

### 2.1. Study Patients

  Inclusion criteria were as follows: (1) vertebral compression fractures from L1 to L5; (2) less than 50% loss of vertebral height; (3) bone mineral density (BMD) of −2.5 or lower; (4) on magnetic resonance imaging (MRI), the fractured vertebral body showed a hypointense signal on T1-weighted images and hyperintense signal on T2-weighted images; and (5) able to tolerate local infiltration anesthesia and to lie prone or laterally for 30 minutes without serious underlying diseases.
  Exclusion criteria were as follows: (1) History of malignancy, infection, or tumor; (2) spinal cord compression or stenosis of the vertebral canal >30% of the local canal diameter; (3) neurologic deficits; (4) uncorrectable bleeding disorders; and (5) severe comorbidity in the heart, lung, or kidney or other serious underlying diseases resulting in intolerance to surgery.

There were 91 patients (age range from 61 to 89 years, mean of 75.75 ± 7.03 years) who underwent vertebral compression fracture treatment. A total of 101 vertebral compression fractures were treated by the modified transverse process-pedicle approach in the authors' institution between June 2016 and September 2018. The locations of the collapsed lumbar vertebrae were as follows: L1, 2 in 5 cases; L1, 3 in 3 cases; L2, 3 in 2 cases; L1 in 31 cases; L2 in 27 cases; L3 in 15 cases; L4 in 5 cases; and L5 in 3 cases. Among the 91 patients enrolled in this study, 22 had sprains, 11 had car accident-related injuries, 37 had fall-related injuries, and the other 21 cases had no definite trauma history.  Table 1 summarizes the detailed characteristics of the patients.

All patients underwent preoperative imaging assessment using a combination of conventional radiography, MRI, and computed tomography (CT). Surgical indications were high signal intensity in the fat-lipid suppression phase on MRI and definite clinical symptoms of sustained severe lumbar and back pain. Out-of-bed activity was allowed 6 hours after the operation. Antiosteoporotic drugs (bisphosphonates, calcitriol, and vitamin D) were prescribed for at least 6 months after the operation.

### 2.2. Surgical Management

**Figure 1:**
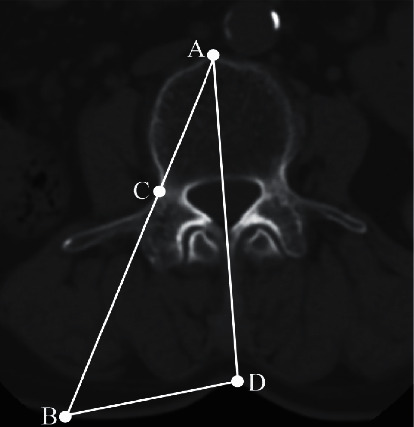
The skin entry point design for unilateral extrapedicular PVP. The skin entry point was determined from the axial image of preoperative CT at the target level. Point A is the junction point of the midline and the anterior edge of the vertebral body. Point D is the junction point of the midline and the skin. Point C is the entry point of the vertebral body. Point B is the junction point of the AC line and the skin, which is also the skin entry point of the unilateral extrapedicular PVP.

**Figure 2:**
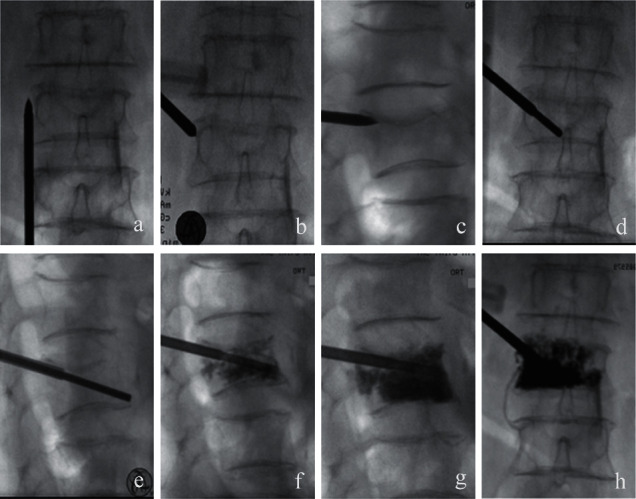
Anteroposterior and lateral views of the needle trajectory inserted into the vertebral body via a unilateral extrapedicular puncture method. (a) Anteroposterior radiograph showed the left transverse process of the fractured vertebral body. (b, c) The needle tip of the bone entry point was located at the bone groove at the junction between the pedicle and the vertebral transitional location. (d, e) Anteroposterior and lateral radiographs showed the optimal position of the working cannula. (f, g) The position of the cement cannula could be adjusted according to the dispersion of cement during the operation. (h) Anteroposterior radiograph showed the bone cement distribution.

The surgical procedure was performed under local infiltration anesthesia with the patient in the prone or lateral position. C-arm fluoroscopy was used for simultaneous viewing of anteroposterior and lateral projections of the spine to identify the point of the vertebral body. Lidocaine (2%) was injected into the skin, lumbar fascia, and deep soft tissues. A 5 mm skin incision was performed at point B (Figure 1). The left transverse process of the fractured vertebral body was located under C-arm fluoroscopy (Figure 2(a)). The needle punctured the transverse process along the BA trajectory, overstrode the superior margin of the transverse process, and proceeded forward, scratching the lateral cortex of the pedicle. During this puncture process, the craniocaudal angle of the needle was increased to reduce the risk of damage to the dural sac and the traveling nerve root and the extraversion angle was increased to avoid injuring the paraspinal venous plexus. After reaching the hard and smooth lateral wall of the pedicle, the needle was slid forward and the needle tip was then stuck in the depressed bone groove at the superolateral junction between the pedicle and the vertebral transitional location. Anteroposterior and lateral views were essential for identifying the optimal position of the needle tip (Figures 2(b) and 2(c)). When the needle continued to penetrate the cortex, the occipital core was pulled out, the bone drill was inserted, and the drill was advanced until the end of the drill was placed in the anterior cortex of the vertebral body. Lateral views confirmed that the midline of the vertebral body was reached or exceeded. The bone drill was then replaced with a working cannula (Figures 2(d) and 2(e)). After successful puncture, bone cement was slowly injected into the vertebral body under C-arm monitoring (Figures 2(f)–2(h)).

### 2.3. Clinical and Radiographic Assessments

The VAS and ODI were recorded preoperatively at 1 day and 6 months postoperatively. Lumbar and back pain was assessed by the VAS. Function recovery was assessed by the ODI. The quantity of injected bone cement, incidence of leakage of bone cement, and other complications were recorded during the surgery. The anterior vertebral height was measured and compared according to preoperative and postoperative imaging (1 day and 6 months).

### 2.4. Statistical Analysis

Statistical analyses were performed using SPSS version 23.0 statistical software. All the measurement data were presented as the mean ± standard deviation $\left(\bar{x}\pm s\right)$. Preoperative and postoperative values between different subgroups were compared using the one-way ANOVA. Differences were considered significant at *P* < 0.05.

## 3. Results

All patients were followed up for 6–12 months, with an average 8.55 ± 1.47 months. The VAS pain and ODI scores at day one (1.63 ± 0.74, 19.70 ± 2.85) after operation were significantly lower than the preoperative scores (7.23 ± 0.79, 40.12 ± 3.92) (*P* < 0.01), but there were no significant differences with the scores at six months (1.52 ± 0.79, 18.84 ± 2.46) (*P* < 0.05) after operation. The anterior vertebral height at day one (24.77 ± 6.02) after surgery was slightly higher than that before surgery (23.86 ± 6.15), but there was no significant difference (*P* < 0.05). The anterior vertebral height at six months (24.14 ± 5.72) after surgery was slightly lower than that at day one after surgery, but there was no significant difference (*P* < 0.05) (Table 2).

**Figure 3:**
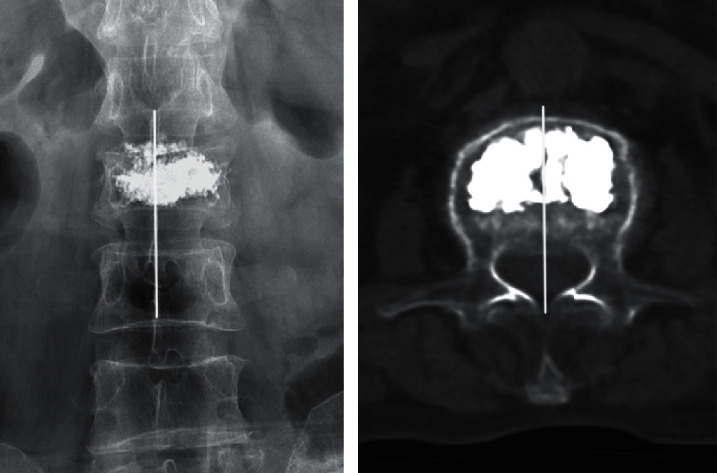
X-ray and CT showed that the distribution of bone cement crossed the midline with satisfactory diffusion.

**Figure 4:**
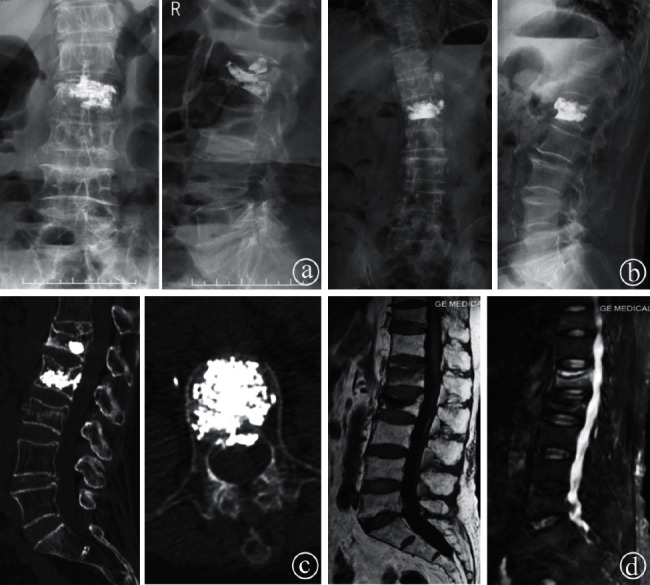
Images of bone cement leakage and adjacent level fracture in PVP cases. X-rays showing that the bone cement had leaked into the intervertebral disk (a). The anterior edge of the vertebral body (b). CT showing that the bone cement had leaked into the vertebral canal (c). MRI scan with compression fracture of the L1 vertebra after PVP of the L2 vertebra (d).

All patients were successfully treated using the modified extrapedicular technique without any recognized clinical complications. The average operation time and the mean volume of the injected cement in a single level were 20.22 ± 4.51 min and 6.04 ± 0.98 mL, respectively. Radiologic outcomes in all fractured vertebrae showed that the diffusion of bone cement could exceed the midline of the vertebral body (Figure 3). Postoperative bone cement leakage was found in 8 patients in the current study. The bone cement leaked into the intervertebral space in 4 cases, the anterior edge of the vertebral body in 3 cases, and the vertebral canal in 1 case, without obvious clinical symptoms. Refracture of the adjacent vertebral body occurred in 1 case at 2 weeks after the surgery (Figure 4).

## 4. Discussion

In relative terms, bilateral PVP showed increased operation time and injected cement volume, while unilateral PVP showed reduced operation time, surgery-related complications, and radiation exposure. In recent years, unilateral PVP has been increasingly used in surgery, resulting in reductions in exposure time to radiation, risk of cement leakage, and complications [2, 10, 11]. Soon et al. found that unilateral PVP using a modified surgical instrument with a directional needle was an excellent example of advancement and refinement in spinal surgery without increased clinical risk and the novel directional needle technique can potentially provide better radiological outcomes than a straight needle [12]. The unilateral extrapedicular needles, advanced through the costopedicular joint, had proper bilateral cement diffusion in the treatment of lumbar vertebral compression fractures [13]. Beall et al. reported an effective extrapedicular modified inferior endplate access to the vertebral body for lumbar vertebral compression fractures. The entry point of the needle was 0.5–1.0 cm above the inferior endplate anterior to the ipsilateral pedicle [14]. However, these techniques were generally complex and required repeated fluoroscopy, which also increased the patients' pain.

**Figure 5:**
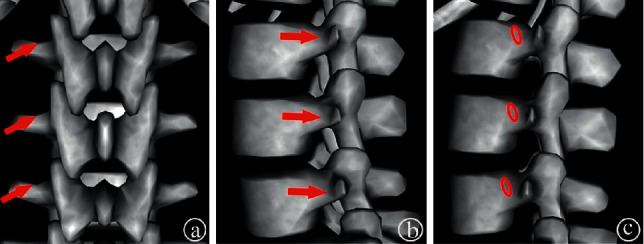
The three bony markers. (a) The superior margin of the transverse process (red arrow). (b) The outer wall of the pedicle (red arrow). (c) The posterolateral cortex of the vertebral body (red circle).

The modified transverse process-pedicle extrapedicular pathway in this study had the following advantages: (1) There was improved safety of the operation. This new technology can be applied to puncture the working cannula from the bottom of the “Kambin” triangle [15] to the contralateral fractured vertebral body. The wide and safe margin from the dural sac and nerve root of the triangle can reduce the risk of intraoperative nerve injury. In addition, the bone entry point of this approach was located in the safe puncture zone of extrapedicular vertebroplasty of lumbar vertebral bodies and was more superior to the midline of the pedicle, reducing the risk of segmental vertebral body artery injury [16]. (2) It alleviated patient pain. Because the puncture path of this technique is within the soft tissue, good local infiltration of anesthesia can be carried out, which can alleviate patient pain during the puncture process. (3) The operative procedure was simplified and controllable. This extrapedicular puncture technique had three definite bony markers (Figure 5), the upper edge of the transverse process, the outer wall of the pedicle, and the posterolateral cortex of the vertebral body, all of which had obvious landmarks, which made it possible to complete the single puncture with the use of C-arm guided fluoroscopy. Moreover, this extrapedicular puncture technique was free from the constraints of a pedicle. During the operation, the direction and depth of the cement cannula can be adjusted flexibly, which made it possible to ensure the ideal dispersion of the cement. (4) Different positions can be maintained according to the patient's condition. The puncture technique was easy to perform because of its clear bony markers. It can be used in prone, lateral, and semilateral positions according to the patient's condition and was especially suitable for patients with poor pulmonary function or those who are unable to lie prone due to pain.

This technique was mainly suitable for L1-3 vertebral fractures, and the degree of fracture compression was less than 50%. Because the puncture path was at the upper edge of the transverse process, the puncture point of the vertebral body was slightly higher than that of the pedicle puncture and it was difficult to penetrate the anterior part of the vertebral body, which limited the application of this technique in patients with vertebral compression degrees greater than 50%. The L4 vertebral body, and especially the L5 vertebral body, are essentially half-elliptic. The flattening of the vertebral body resulted in the difficulty of detecting the third bony marker, which limited the application of the puncture technique to a certain extent. For L4 and L5 vertebral fractures, the shape of the vertebral body should be judged by preoperative CT examination, and then the surgeon decides whether the puncture technique should be applied or not be applied. In patients with hypertrophy and a high position of the transverse process, the adjustment of the sagittal puncture angle was limited. It was difficult to puncture the cement cannula to the ideal position of the vertebral body. If the diffusion effect of unilateral puncture was poor, a contralateral pedicle puncture was necessary.

There were some limitations in this study. The number of patients included in this study was relatively small. The follow-up period was relatively short. In addition, no control group was established in this study. Further long-term follow-up studies in a large patient population are warranted to generalize our results.

## 5. Conclusion

A needle trajectory with a modified transverse process-pedicle approach can be easily and precisely planned using unambiguous anatomical landmarks under fluoroscopic guidance, enabling sufficient bone cement distribution and tremendous pain relief.

## Figures and Tables

**Table 1:** Characteristics of the study patients.

Characteristics	Patients
Case	91
Age (years)	75.75 ± 7.03
Sex (male/female)	21/70
BMD T-score	3.53 ± 0.61
Follow-up duration (months)	8.55 ± 1.47

BMD, bone mineral density.

**Table 2:** Changes in VAS and ODI scores and anterior vertebral height during follow-up periods.

Parameters	Preop	1 day postop	6 months postop	*P*_1_	*P*_2_
VAS (*n*_1_ = 91)	7.23 ± 0.79	1.63 ± 0.74	1.52 ± 0.79	＜0.01	0.34
ODI (*n*_1_ = 91)	40.12 ± 3.92	19.7 ± 2.85	18.84 ± 2.46	＜0.01	0.084
Anterior vertebral height (*n*_2_ = 101)	23.86 ± 6.15	24.77 ± 6.02	24.14 ± 5.72	0.56	0.779

VAS, visual analogue scale; ODI, Oswestry disability index; *P*_1_, preoperative vs. postoperative day 1; *P*_2_, postoperative day 1 vs. postoperative month 6; *n*_1_, total number of patients; *n*_2_, total number of vertebrae.

## Data Availability

The data generated in this study are available from the corresponding authors upon request.

## References

[1] Malik A. T., Retchin S., Phillips F. M. (2020). Declining trend in osteoporosis management and screening following vertebral compression fractures-a national analysis of commercial insurance and medicare advantage beneficiaries. *The Spine Journal*.

[2] Yang S., Chen C., Wang H., Wu Z., Liu L. (2017). A systematic review of unilateral versus bilateral percutaneous vertebroplasty/percutaneous kyphoplasty for osteoporotic vertebral compression fractures. *Acta Orthopaedica et Traumatologica Turcica*.

[3] Wu Z., Linqiang Y., Mo L., Jiang X., Cui J., Feng Y. (2021). “Comparison of cement leakage rate and severity after percutaneous vertebroplasty for osteoporotic vertebral compression fractures using front-opening versus side-opening cannulas. *Orthopedics*.

[4] Griffoni C., Lukassen J. N. M., Babbi L. (2020). Percutaneous vertebroplasty and balloon kyphoplasty in the treatment of osteoporotic vertebral fractures: a prospective randomized comparison. *European Spine Journal*.

[5] Jurczyszyn A., Czepko R., Banach M., Godlewski B., Masłowski P., Skotnicki A. (2015). Percutaneous vertebroplasty for pathological vertebral compression fractures secondary to multiple myeloma-medium-term and long-term assessment of pain relief and quality of life. *Advances in Clinical and Experimental Medicine*.

[6] Ng P. P., Caragine L. P., Dowd C. F. (2002). Percutaneous vertebroplasty: an emerging therapy for vertebral compression fractures. *Seminars in Neurology*.

[7] Nieuwenhuijse M. J., Muijs S. P. J., van Erkel A. R., Dijkstra S. P. D. (2010). A clinical comparative study on low versus medium viscosity PolyMethylMetAcrylate bone cement in percutaneous vertebroplasty. *Spine*.

[8] Kim B. S., Hum B., Park J. C., Choi I. S. (2014). Retrospective review of procedural parameters and outcomes of percutaneous vertebroplasty in 673 patients. *Interventional Neuroradiology*.

[9] Zhang L., Liu Z., Wang J. (2015). Unipedicular versus bipedicular percutaneous vertebroplasty for osteoporotic vertebral compression fractures: a prospective randomized study. *BMC Musculoskeletal Disorders*.

[10] Chen Y. C., Zhang L., Li E. N. (2019). Unilateral versus bilateral percutaneous vertebroplasty for osteoporotic vertebral compression fractures in elderly patients: a meta-analysis. *Medicine*.

[11] Wang W., Duan K., Ma M. (2019). Can an unipedicular approach replace bipedicular percutaneous vertebroplasty for osteoporotic vertebral compression fracture?. *Journal of Back and Musculoskeletal Rehabilitation*.

[12] Soon W. C., Mathew R. K., Timothy J. (2015). Comparison of vertebroplasty using directional versus straight needle. *Acta Radiologica Open*.

[13] Cianfoni A., Massari F., Ewing S., Persenaire M., Rumboldt Z., Bonaldi G. (2014). Combining percutaneous pedicular and extrapedicular access for tumor ablation in a thoracic vertebral body. *Interventional Neuroradiology*.

[14] Beall D. P., Parsons B., Burner S. (2016). Technical strategies and anatomic considerations for an extrapedicular modified inferior endplate Access to thoracic and lumbar vertebral bodies. *Pain Physician*.

[15] Ozer A. F., Suzer T., Can H. (2017). Anatomic assessment of variations in kambin’s triangle: a surgical and cadaver study. *World neurosurgery*.

[16] Heo D. H., Cho Y. J. (2011). Segmental artery injury following percutaneous vertebroplasty using extrapedicular approach. *Journal of Korean Neurosurgical Society*.

